# Serum Levels of Choline-Containing Compounds Are Associated with Aerobic Fitness Level: The HUNT-Study

**DOI:** 10.1371/journal.pone.0042330

**Published:** 2012-07-30

**Authors:** Anja Bye, Riyas Vettukattil, Stian T. Aspenes, Guro F. Giskeødegård, Ingrid S. Gribbestad, Ulrik Wisløff, Tone F. Bathen

**Affiliations:** 1 Department of Circulation and Medical Imaging, Norwegian University of Science and Technology, Trondheim, Norway; 2 K. G. Jebsen Center of Exercise in Medicine, Trondheim, Norway; Instituto de Investigación Sanitaria INCLIVA, Spain

## Abstract

**Background:**

Cardiovascular disease (CVD) is a leading cause of death worldwide, and the number of people at risk is continuously growing. New methods for early risk prediction are therefore needed to actuate prevention strategies *before* the individuals are diagnosed with CVD. Several studies report that aerobic fitness level, measured as maximal oxygen uptake (VO_2max_), is the single best predictor of future CVD mortality in healthy people. Based on this, we wanted to study differences between healthy individuals with a large difference in VO_2max_-level to identify new biomarkers of low aerobic fitness that may also have potential as early biomarkers of CVD risk.

**Methodology/Principal Findings:**

Serum samples from 218 healthy individuals with a low VO_2max_ (n = 108, 63 women) or high VO_2max_ (n = 110, 64 women) were analysed with MR metabolomics. In addition, standard clinical-chemical analyses for glucose, lipids, liver enzymes, micro-CRP, and colorimetric analysis on circulating choline were performed. Individuals in the low VO_2max_-group had increased serum levels of free choline, decreased phosphatidylcholine, increased glucosę and decreased unsaturated fatty acids compared to the individuals in the high VO_2max_–group.

**Conclusions/Significance:**

Aerobic fitness dependent differences in serum levels of free choline and phosphatidylcholine are observed. They should be further studied as potential early markers of CVD risk.

## Introduction

Cardiovascular disease (CVD) is a leading cause of death worldwide, and the number of people at risk is continuously growing [1]. New methods for early risk prediction are therefore needed to actuate prevention strategies *before* the individuals are diagnosed with CVD. Aerobic fitness level, measured as maximal oxygen uptake (VO_2max_), is a strong marker for cardiac health. Large-scale epidemiological studies have demonstrated that low VO_2max_ is the single best predictor of future CVD mortality both in healthy individuals and in patients with CVD [2]–[6]. Based on this, more knowledge of the differences between healthy individuals with a large difference in VO_2max_-level will be of great interest to identify new biomarkers of low aerobic fitness that may also have a potential as an early biomarker of CVD risk.

Emerging metabolite profiling technologies have recently made it possible to acquire “snapshots” of the metabolic processes at a given point in time [7], [8]. This methodology, termed metabolomics, involves a high throughput analysis of small-molecular metabolites that are downstream products of preceding gene expressions and protein activity. Within systems biology, magnetic resonance (MR) metabolomics has become one of the key platforms, allowing rapid analysis of samples with minimal sample preparation. The acquired metabolic profiles can be useful for a better understanding of the metabolic perturbations associated with health and disease.

Previously, serum and plasma MR metabolomics have been successfully used in the detection of biomarkers associated with various clinical conditions such as coronary artery disease and myocardial infarction [9]–[11]. Serum metabolites such as citric acid, threonine, and choline have previously been associated with the incidence of CVD, but so far the evidence is sparse [9]–[11]. To our knowledge, no previous study has searched for serum metabolites associated with aerobic fitness level in a healthy population. The aim of the present study was to investigate metabolic differences between healthy individuals with high and low VO_2max_ by MR metabolomics, and further to describe these differences qualitatively and quantitatively.

## Results

The high and the low VO_2max_-groups, which were matched for age, fasting time and level of self-reported physical activity, had significantly different body weight, waist circumference, waist-to-hip-ratio, body mass index (BMI), mean arterial blood pressure, resting heart rate, non-fasting glucose, triglycerides, micro C-reactive protein (CRP), alanine aminotransferase (ALAT) and gamma glutamyl transferase (Gamma-GT), but not total cholesterol (Table 1). Data from questionnaires revealed that the own-reported health status was significantly better in the high VO_2max_-group compared to the low VO_2max_-group (Table 2). Dietary questionnaires indicated only differences in fruit and berry intake between the two groups (Table 2).

**Table 1:** A statistical overview of the participants in this study.

Variable	Low VO_2max_-group			High VO_2max_-group			p-value
	n	mean	CI	n	mean	CI	
Age	108	49.5	48.4–50.6	110	49.5	48.4–50.6	–
VO_2max_ (mL⋅kg^−0.75^⋅min^−1^)	108	93.9	90.9–96.9	110	138.0	133.4–142.7	–
Physical activity index score	108	3.7	3.4–4.0	110	3.7	3.4–4.0	–
Waist (cm)	108	93.6	91.5–95.7	110	86.3	84.6–88.1	0.0004**
Hip (cm)	108	103.8	102.4–105.3	110	100.1	99.2–101.1	0.0004**
Arm circumference (cm)	108	30.3	29.7–30.9	110	28.7	28.3–29.2	0.0004**
Weight (kg)	108	80.7	77.9–83.5	110	73.5	71.4–75.6	0.0004**
Waist-to-hip-ratio	108	0.90	0.88–0.91	110	0.86	0.85–0.87	0.0004**
BMI	108	27.5	26.8–28.3	110	24.8	24.3–25.2	0.0004**
Heart rate at rest	103	62.3	60.2–64.4	102	55.8	53.9–57.7	0.0004**
Systolic blood pressure (mmhg)	108	128.5	125.6–131.5	110	124.9	122.2–127.5	0.070
Diastolic blood pressure (mmhg)	108	75.2	73.2–77.1	110	72.5	70.7–74.3	0.051
Mean arterial pressure	108	93.0	90.9–95.1	110	90.0	88.0–91.9	0.038*
Alanine aminotransferase (U/L)	13	40.3	20.7–59.9	25	25.3	21.3–29.4	0.038*
Gamma glutamyl transferase (U/L)	13	52.8	20.2–85.4	25	27.8	18.8–36.8	0.049*
Non-fasting glucose (mmol/L)	103	5.7	5.4–6.0	104	5.2	5.0–5.3	0.004**
Cholesterol (mmol/L)	103	5.6	5.4–5.7	104	5.5	5.3–5.6	0.447
HDL-cholesterol (mmol/L)	103	1.4	1.3–1.4	104	1.5	1.4–1–5	0.074
Triglycerides (mmol/L)	77	1.7	1.5–1.9	90	1.3	1.1–1.4	0.002**
Serum micro C-reactive protein (mg/L)	76	2.2	1.3–3.2	90	1.2	0.9–1.6	0.040*

VO_2max_: Maximal oxygen uptake, CI: Confidence Interval, BMI: Body Mass Index, HDL: High Density Lipoprotein. P-values below 0.05 are flagged. **p<0.005, *p<0.05.

**Table 2:** Data from questionnaires.

Variable	Low VO_2max_-group			High VO_2max_-group			p-value
	n	mean	CI	n	mean	CI	
Own reported health status (scale 1–4)	104	3.0	2.8–3.1	107	3.3	3.2–3.4	0.001**
Vegetables intake (scale 1–5)	108	3.5	3.3–3.6	110	3.6	3.4–3.7	0.293
Fruit and berry intake (scale 1–5)	108	3.4	3.2–3.7	110	3.8	3.6–4.0	0.029*
Sausage and hamburger intake (scale 1–5)	108	1.3	1.3–1.5	108	1.2	1.2–1.4	0.102
High-fat fish intake (scale 1–5)	108	1.5	1.5–1.8	108	1.5	1.4–1.7	0.730

Food intake: 1 = 0–3 times a month, 2 = 1–3 times a week, 3 = 4–6 times a week, 4 = Once a day, 5 = 2 times or more each day. VO_2max_: Maximal oxygen uptake, CI: Confidence interval. P-values below 0.05 are flagged. **p<0.01, *p<0.05.

**Figure 1:**
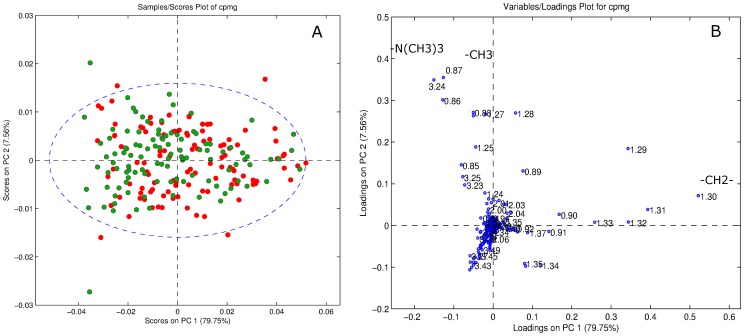
Principal Component Analysis (PCA) of the serum 1H MR spectra. (A) In the score plot, high VO_2max_ subjects are shown in green (higher density in upper left part) and low VO_2max_ subjects are shown in red. (B) The loadings plot visualizes the differences in metabolites between the two groups. The signals originating from within the core of the serum lipoprotein particles (-CH_3_ at 0.86 ppm, -CH_2_- at 1.3 ppm) and choline-containing compounds (-N (CH_3_)_3_^+^, at 3.24 ppm) are mainly responsible for the clustering. VO_2max_: Maximal oxygen uptake.

**Figure 2:**
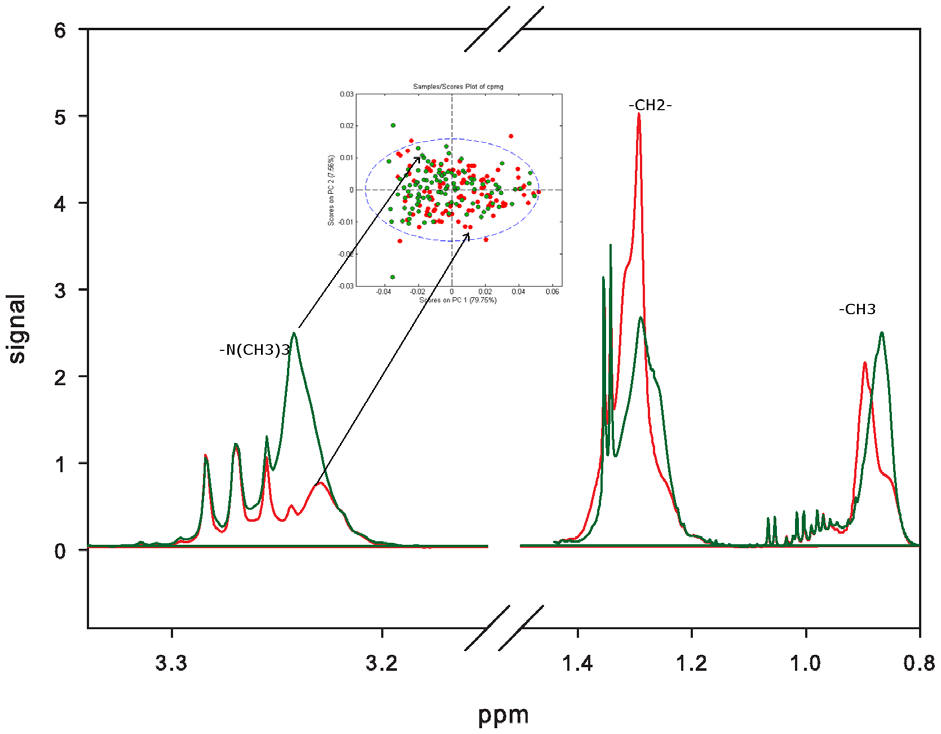
Visualization of the metabolic differences in MR spectra. The green spectrum is from a high VO_2max_ subject (green dots in PCA score plot) and the red spectrum is from the low VO_2max_ (red dots in PCA score plot). VO_2max_: Maximal oxygen uptake.

MR spectra indicated differences between the metabolic profiles of the high and low VO_2max_-groups (Figure 1A). Exploration of the corresponding loading profiles (Figure 1B) and MR spectra (Figure 2) showed that low VO_2max_-subjects had higher levels of lipid methylene (-CH2-) protons (peak at 1.3 ppm), indicating decreased amounts of unsaturated fatty acids in serum from the low VO_2max_-subjects. The low VO_2max_-subjects also had lower levels of phosphatidylcholine (PtdCho) (-N(CH_3_)_3_^+^, peak at 3.24 ppm) (Figure 2). A permutation test showed that the differences in the metabolic profiles between high and low VO_2max_-subjects were highly significant (p<0.001). The model created by the MR metabolomics analysis could predict whether a subject has a low or high VO_2max_ with a sensitivity and specificity of 63% and 65%, respectively.

A subsequent colorimetric analysis to further study the differences in choline-containing compounds showed that the levels of free choline were significantly higher in the low VO_2max_-group compared to the high VO_2max_-group (14.57±1.55 vs. 10.13±0.91 µM, p = 0.017). The serum choline levels seemed to correlate with the serum triglycerides levels (high VO_2max_-group, r = 0.50, p<0.005 and low VO_2max_-group, r = 0.74, p<0.0001). There was no correlation between free choline levels and fasting status.

**Figure 3:**
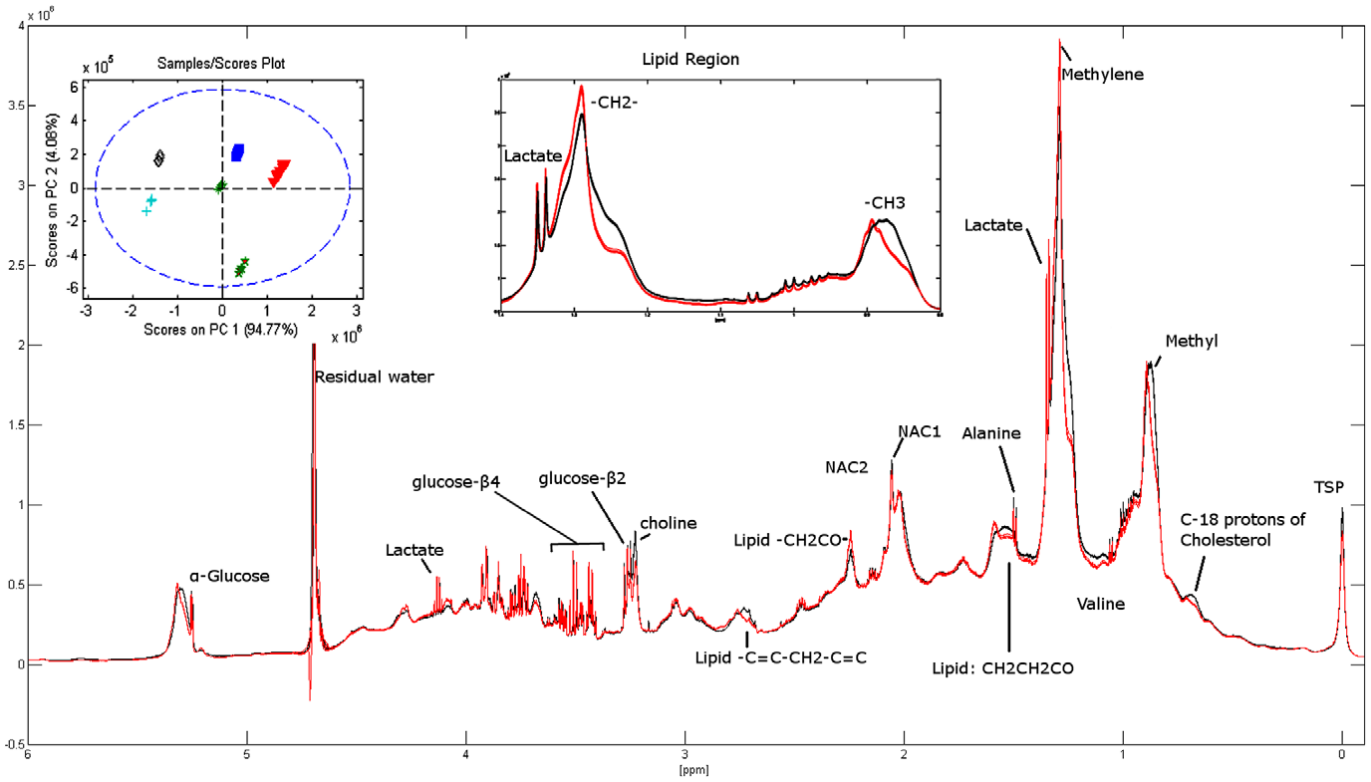
Representative ^1^H NOESYGPPR 1D spectra with assignments of the main metabolites. The spectra in red (and black) consist of 5 spectra (superimposing) from two of the subjects. The reproducibility was evaluated by PCA and the score plot shows the spectra from all 6 subjects, clearly depicting larger inter subject variance compared to intra subject variance. NACl and NAC2 refer to composite acetyl signals from **α**1-acid glycoprotein. PCA: Principal Component Analysis.

Replicate ^1^H MR spectra of serum with assignments of the main metabolites are illustrated in Figure 3. The score plot (Figure 3) clearly displays a larger inter subject variance compared to intra subject variance, which indicates excellent reproducibility. To further assess the agreement between metabolite levels obtained by laboratory assays and MR, the glucose concentration obtained by standard methodology were correlated with the MR signal intensities (relative quantification by peak integration 3.90–3.94 ppm) for glucose. The data showed strong correlations (R^2^ = 0.83).

## Discussion

The main findings of this study were that the subjects with low VO_2max_ had increased serum levels of free choline and decreased serum levels of phosphatidylcholine (PtdCho) compared to subjects with high VO_2max_. In human cells, the majority of choline is taken up by the cells and conversed into PtdCho. Since the ratio of choline/PtdCho is switched between the subjects with high and low VO_2max_, there might be difference in one of the enzymes of the plasma membrane, phospholipase D (PLD). PLD catalyzes the hydrolysis of PtdCho to phosphatic acid (PA), releasing soluble choline. High PLD activity and increased level of PA has previously been associated with oxidative stress, hypoxia, inflammation, atherosclerosis and hypertension (reviewed in [12]). In the heart, high PLD activity and increased level of PA are suggested to be involved in the signaling cascade promoting pathological cardiac hypertrophy [13]. If our assumptions are correct, even healthy individuals with a low aerobic fitness may have a high PLD activity, which may link low aerobic fitness to the future development of CVD.

Other explanation for the high levels of serum choline in the low VO_2max_-group may be release of choline from damaged organs, impaired tissue uptake or choline-rich diet [14], [15]. In patients with severe repetitive arrhythmias and hemodynamic compromises, choline has been shown to leak from ischemic tissues into the blood stream [16]–[18]. However, since the participants in the current study were healthy it seems unlikely that the increased levels of choline arise from myocardial release. Regarding diet, a previous study indicated a weak inverse association between serum choline and time since last meal [19]. In the current study the groups were matched on fasting status, and no important differences were found in diet. Thus, the observed differences in free choline levels are not likely to be caused by differences in food intake.

High serum levels of free choline have previously been associated with an increased prevalence of the metabolic syndrome (a cluster of risk factors of CVD) [19]. Since the subjects in the low VO_2max_-group not fulfilled the criteria for the metabolic syndrome, our results may indicate that serum levels of choline may have prognostic value for future CVD even among healthy subjects. Free choline levels should therefore be assessed in a large healthy cohort to prospectively study the prognostic value for later cardiovascular events. Furthermore, elevated levels of choline have recently been recognized as a novel biomarker for early risk stratification in patients with suspected acute coronary syndrome [16]–[18]. To our knowledge, no previous study has reported associations between VO_2max_ level and free choline.

In addition to the differences in choline-containing compounds, the MR spectra also indicated that the subjects with low VO_2max_ had decreased amounts of unsaturated fatty acids. Decreased serum levels of unsaturated fatty acids have previously been associated with increased risk of CVD [20].

The differences in weight, waist circumference, waist-to-hip-ratio, BMI, mean arterial blood pressure, resting heart rate, non-fasting glucose, triglycerides, micro-CRP between the high and low VO_2max_-groups are supported by previous findings [21]–[23]. In addition, the increased levels of circulating liver enzymes (ALAT and Gamma-GT) in the low VO_2max_-group may reflect more liver fat and increased insulin resistance [24]. Previous results from a study of rats with genetically low aerobic capacity indicate that low VO_2max_ impairs the hepatic oxidative capacity and therefore contributes to increased amounts of liver fat [25]. Elevated levels of Gamma-GT, even within the normal range, have previously been associated with the presence of CVD risk factors, metabolic syndrome, and type 2-diabetes [26], [27].

Ideally, obtaining blood samples after overnight fasting would be preferable due to the elimination of chylomicrones from the circulation further enabling a more detailed overview of lipids and lipoprotein sub-classes [28]. However, fasting for several hours is neither easy to accomplish in a population-based large-scale study, nor preferable when performing a VO_2max_-test. In addition to a possible influence on the lipid metabolites in the MR spectra, it is also possible that the lack of fasting may have interfered with the results on glucose and total cholesterol levels. In this study there was no difference in total cholesterol levels between the high and low VO_2max_-group. Previous studies have reported inverse correlation between VO_2max_ and total cholesterol [29], [30].

In conclusion, low VO_2max_ is associated with elevated levels of free choline and decreased levels of phosphatidylcholine, even in a cohort of healthy individuals. The precise reason for the shift in the choline/phosphatidylcholine ratio between subjects in the high versus the low VO_2max_-group is unclear, but might be associated with phospholipase activity, or differences in cardiac or hepatic release. Further studies should be conducted on free choline and phosphatidylcholine to validate their potential as early risk-markers of CVD and predictors of VO_2max_.

## Materials and Methods

### Study Participants

The third wave of the Nord-Trøndelag Health Study (HUNT3) in Norway was carried out between 2006 and 2008 and the results reported in the present publication stems from this part of the large HUNT study. Among 50,821 participants in HUNT3, 4631 healthy, adult subjects attended a sub-study designed to measure VO_2max_, called the Fitness Study [31]. Participants in the Fitness Study reported to be free from heart- or lung-disease (details previously described [31]). From the Fitness Study-population, 220 individuals between 40 and 59 years were selected pair-wise with one having low and the other high VO_2max_ (selected from top or bottom 15 subjects within each age-year), but otherwise same gender, equal age in years, same physical activity index score (within 15% difference) and equal time since last meal. Subjects were ranged according to VO_2max_ reported as mL⋅kg^−0.75^⋅min^−1^, and maximum five pairs of subjects were matched from each age-year. Two subjects did not provide a blood sample, and the study thus included 218 subjects (45 males and 63 females in the low VO_2max_-group, and 46 males and 64 females in the high VO_2max_-group).

The study was approved by the Regional Committee for Medical Research Ethics, the Norwegian Data Inspectorate, and by the National Directorate of Health. The study is in conformity with Norwegian laws and the Helsinki declaration, and all participants signed a document of consent.

### Clinical Measurements

Weight and height were measured on a combined scale (Model DS-102, Arctic Heating AS, Nøtterøy, Norway), and BMI was calculated as weight divided by height squared (kg m^−2^). Blood pressure and resting heart rate were both measured while sitting (Critikon Dinamap 845XT, GE Medical Systems, Little Chalfont, Buckinghamshire, United Kingdom) and followed established guidelines [32].

An individualized protocol was applied to measure VO_2max_ treadmill running to exhaustion [33]. The VO_2max_-test was performed using a ramp protocol where the speed was constant and the incline was increased with 2% every second minute until VO_2max_ was reached. Oxygen uptake kinetics were measured directly by a portable mixing chamber gas-analyzer (Cortex MetaMax II, Cortex, Leipzig, Germany) with the participants wearing a tight face mask (Hans Rudolph, Kansas City, USA) connected to the MetaMax II. The system has previously been found valid [34]. Heart rate was measured by radio telemetry (Polar S610i, Polar Electro Oy, Kempele, Finland). From the warm-up pace, the load was regularly increased when oxygen uptake kinetics flattened. Along with a respiratory quotient of 1.05 or higher, a maximal test was considered achieved when the oxygen uptake did not increase more than 2 mL⋅kg^−1^⋅min^−1^ at the highest effort or before the participant disembarked the treadmill [35]. VO_2max_ was measured as litres of oxygen per minute (L⋅min^−1^), and subsequently calculated as VO_2max_ relative to body mass (mL⋅kg^−1^⋅min^−1^) and VO_2max_ scaled (mL⋅kg^−0.75^⋅min^−1^).

### Blood Analysis

All clinical-chemical analyses were performed on fresh venous non-fasting blood samples at Levanger Hospital, Norway. Non-fasting glucose (mmol/L) was analysed by Hexokinase/G-G-PDH methodology (reagent kit 3L82-20/3L82-40 Glucose, Abbot, Clinical Chemistry, USA). HDL-cholesterol (mmol/L) was analysed by Accelerator selective detergent methodology (reagent kit 3K33-20 Ultra HDL, Abbot, Clinical Chemistry, USA). Triglycerides (mmol/L) were analysed by Glycerol Phosphate Oxidase methodology (reagent kit; 7D74 Triglyceride, Abbot, Clinical Chemistry, USA). Creatinine (mg/dl) was analysed by Alkaline Picrate methodology (reagent kit; 7D65-20 Creatinine, Abbot, Clinical Chemistry, USA). Alanine aminotransferase (U/L) was analysed by NADH (with P-5′-P) methodology (reagent kit; 8D36-30 Alanine aminotransferase activated, Abbot, Clinical Chemistry, USA). Measurements below the instrument range were recorded as 9 U/L. Gamma glutamyl transferase (Gamma-GT) (U/L) was analysed by L-Gamma-glytamyl-3-carboxy-4-nitroanilide substrate methodology (reagent kit; 7D65-20 Gamma-glutamyl transferase, Abbot, Clinical Chemistry, USA). Measurements below and above instrument range were recorded as 3 U/L and 1544 U/L, respectively. Serum micro C-reactive protein (mg/L) was analysed by Areoset CRP Vario kit (Abbot, Clinical Chemistry, USA). Measurements below instrument range are recorded as 0.

### Questionnaire-based Information

Physical activity was registered based on the responses to a self-administered questionnaire applied (http://www.ntnu.edu/hunt/data/que) [36]. The questionnaires included three questions: Question 1: “How frequently do you exercise?”, with the response options “Never” (0), “Less than once a week” (0), “Once a week” (1), “2–3 times per week” (2.5) and “Almost every day” (5). Question 2: “If you exercise as frequently as once or more times a week: How hard do you push yourself?” with the response options: “I take it easy without breaking a sweat or losing my breath” (1), “I push myself so hard that I lose my breath and break into sweat” (2) and “I push myself to near exhaustion” (3). Question 3: “How long does each session last?”, with the response options: “Less than 15 minutes” (0.1), “16–30 minutes” (0.38), “30 minutes to 1 hour” (0.75) and “More than 1 hour” (1.0). Each participant’s response to the above mentioned three questions (i.e. numbers in brackets) were multiplied to calculate a physical activity index score [36]. As the second and third question only addressed people who exercised at least once a week, both “Never” and “Less than once a week” yielded an index score of zero. Participants with a zero score were categorized as inactive.

Dietary habits were self-reported in a questionnaire. For “Fruit and berries”, “Vegetables”, “Sausages/hamburgers” and “High-fat fish” the possible response-options were “0–3 times a month” (1), “1–3 times a week” (2), “4–6 times a week” (3), “once a day” (4), and “twice or more a day” (5). Health-status was also self-reported and the options were “bad” (1), “not quite good” (2), “good” (3), or “very good” (4). The mean values were calculated from the answers from all the participants in each group (i.e. numbers in brackets).

### Metabolic Profiling

Venous non-fasting blood samples were collected in serum-tubes with no additives. The blood was centrifuged at 3000 rpm for 10 minutes approximately 1 hour after sample collection. The serum samples were stored at −80°C in the biobank until being used for metabolic profiling. The serum samples were slowly thawed at 4°C. Aliquots of 150 µL were mixed with equal amounts of buffer solution (Na_2_HPO_4_ ×7H_2_O (0.075M), 4% NaN_3_ in H_2_O (5ml, mass % of NaN_3_ versus mass % of H_2_O), TSP (3-(trimethyl-silyl) propionic acid-d4, 0.4g), D_2_O (100 mL), pH adjusted to 7.4 with 1M HCl (1M NaOH), filled up to 500 mL with H_2_O) and transferred to high-quality 3 mm MR tubes. The ratio between H_2_O and D_2_O was 90∶10 in all samples. In order to assess the reproducibility of sample preparation and spectral acquisition, our daily protocol included a set of five samples individually prepared from a single healthy individual every day.

### MR Experiments

The MR spectra were acquired using a Bruker Avance II (Bruker Biospin, Rheinstetten, Germany) with digital receiver unit (DRU) operating at 600 MHz for proton (1H). The probe was a TCI 1H-13C/15N/D with z-gradient and automated tuning and matching unit. All spectra were recorded in an automatic fashion using a BACS-60 sample changer and the ICON-NMR software (Bruker Biospin). Proton spectra were obtained at a constant temperature of 310 K using a modified Carr-Purcell-Meiboom-Gill (CPMG) pulse sequence with presaturation during the relaxation delay (Bruker: cpmgpr1d) to achieve water suppression and to facilitate the detection of low molecular weight species by avoiding the large overlapped signals derived from proteins and large molecules. The spectra were collected with 64 scans and 4 dummy scans. The acquisition time is set to 3.067 sec, measuring the FID via collection of 36864 complex data point resulting in a sweep width of 20.0363 ppm. A relaxation delay of 4 seconds was used, during which a presaturation of 25 Hz was applied. Effective echo time was 80ms and data acquisition starts at maximum of last echo. An exponential apodization of 1Hz was applied prior to Fourier transform. Measurement and processing was done in full automation using Bruker standard automation programs controlled by ICON-NMR (along with TopSpin v2.1 patchlevel 6). Chemical shift was calibrated to the middle of the alanine peaks at 1.50 ppm. The reproducibility spectra were acquired using nuclear Overhauser effect spectroscopy (NOESY, Bruker: noesygppr1d) with the same parameters as CPMG with the exception of 32 scans. The assignments of chemical shifts were done on the basis of previously published data [37].

### Data Processing and Multivariate Analysis

Data analysis was performed with MATLAB (Version 7.9.0; The Math Works, Natick, MA, USA). The spectra were divided into 850 segments, each 0.01 ppm wide for a spectral window ranging from 0.5 to 9.0 ppm to reduce minor chemical shift alterations [38]. The segments between 4.5–5.0 ppm were excluded to remove variation in water suppression efficiency. Spectra were finally normalized by setting the total spectral area to a constant value ( = 1) for all spectra to minimize possible differences in serum concentration between the samples.

Unsupervised principal component analysis (PCA) and supervised partial least squares discriminant analysis (PLS-DA) were performed using PLS_Toolbox v5.8.3 (Eigenvector Research, Manson, WA, USA). PCA reduces the dimensionality of the data and summarizes the structure of the multiple MR spectra visualized in score plots and loading profiles. The variance structure of the data is explained through linear combinations of the variables called principal components (PCs). The first PCs will be in the direction explaining most of the variance in the data set. In the score plot of the PCs, samples with a similar metabolic profile will cluster, while the corresponding loading profile displays the importance of each variable within the PC. PLS-DA is a supervised classification method which uses the class information to detect variables generating maximum separation between the classes (high and low aerobic capacity). All statistical models were cross-validated with a single 10-fold Venetian blind cross validation; in each run 10% of the data were left out of the training and used to test the model. The optimal model contains the number of latent variables yielding the lowest percentage of misclassification. A permutation test was performed (10000 permutations) to evaluate the significance of the difference between the classes [39].

To evaluate the reproducibility of the sample preparation and metabolomics analyses, PCA of the replicate spectra from single subjects (in total 30 sample preparations from 6 subjects) were performed for comparison of the inter versus intra subject variance.

### Colorimetric Analysis of Free Choline

In a sub-cohort of 39 participants (20 women and 19 men) from the low VO_2max_-group and 38 participants (21 women and 17 men) from the high VO_2max_-group, the level of free choline was measured in serum. The groups were matched on fasting status, age and physical activity index score. The serum was analysed with the Choline/Acetylcholine Quantification Kit according to the manufacturer’s instructions (Abnova, Taipei City, Taiwan).

### Statistical Analyses

PASW Statistics 17.0 (IBM, New York, USA) was used for traditional statistical analyses. All statistical tests were two-sided, and p-values below 0.05 were considered statistical significant. Kolmogorov-Smirnov test was used to test for normality. One-Way ANOVA was used for comparing variables between the high and the low VO_2max_-groups, and Kruskal-Wallis test was used in non-parametric analyses. Results are given in mean ± SE. Pearson’s correlation was used to study associations between normally distributed variables, and Spearman’s correlation was used in non-parametric analyses. The correlation analyses were performed separately for the high and low VO_2max_-group.
